# Synchrotron tomography of magnetoprimed soybean plant root system architecture grown in arsenic-polluted soil

**DOI:** 10.3389/fpls.2024.1391846

**Published:** 2024-07-02

**Authors:** Anis Fatima, Sunita Kataria, Meeta Jain, Rajkumar Prajapati, Lovely Mahawar

**Affiliations:** ^1^ Technical Physics Division, Bhabha Atomic Research Centre, Mumbai, India; ^2^ School of Biochemistry, Devi AhilyaVishwavidyalaya, Indore, MP, India; ^3^ Department of Plant Physiology, Faculty of Agrobiology and Food Resource, Slovak University of Agriculture, Nitra, Slovakia; ^4^ Department of Plant Physiology, Umeå Plant Science Centre, Umeå University, Umeå, Sweden

**Keywords:** synchrotron tomography, magnetopriming, arsenic toxicity, root morphology, soybean

## Abstract

The present study evaluated the repercussions of magnetopriming on the root system architecture of soybean plants subjected to arsenic toxicity using synchrotron radiation source based micro-computed tomography (SR-µCT). This will be used evey where as abbreviation for the technique for three-dimensional imaging. Seeds of soybean were exposed to the static magnetic field (SMF) of strength (200 mT) for 1h prior to sowing. Magnetoprimed and non-primed seeds were grown for 1 month in a soil–sand mixture containing four different levels of sodium arsenate (0, 5, 10, and 50 mg As kg^−1^ soil). The results showed that arsenic adversely affects the root growth in non-primed plants by reducing their root length, root biomass, root hair, size and number of root nodules, where the damaging effect of As was observed maximum at higher concentrations (10 and 50 mg As kg^−1^ soil). However, a significant improvement in root morphology was detected in magnetoprimed plants where SMF pretreatment enhanced the root length, root biomass, pore diameter of cortical cells, root hair formation, lateral roots branching, and size of root nodules and girth of primary roots. Qualitative analysis of x-ray micro-CT images showed that arsenic toxicity damaged the epidermal and cortical layers of the root as well as reduced the pore diameter of the cortical cells. However, the diameter of cortical cells pores in magnetoprimed plants was observed higher as compared to plants emerged from non-primed seeds at all level of As toxicity. Thus, the study suggested that magnetopriming has the potential to attenuate the toxic effect of As and could be employed as a pre-sowing treatment to reduce the phytotoxic effects of metal ions in plants by improving root architecture and root tolerance index. This study is the very first exploration of the potential benefits of magnetopriming in mitigating the toxicity of metals (As) in plant roots utilizing the micro-CT technique.

## Introduction

In recent decades, heavy metal (HM) pollution has emerged as a serious problem for soils and aquatic ecosystems worldwide due to rapidly increasing urbanization and industrialization (Narayanan and Ma, 2023). Among the various stresses [salinity, ultraviolet (UV), drought, HMs, temperature, insects, pests etc.] in the terrestrial environment, HMs are regarded as the chief hazardous culprit due to their highly toxic, irreversible, and enduring nature (Kataria and Jain, 2018; Prajapati et al., 2020; Kashyap et al., 2021). Excessive concentrations of HMs in the soil not only alter its physiochemical properties but also severely impact the plants and animal health (Mahawar et al., 2021). Arsenic (As) is a non-essential noxious metalloid naturally present in the soil (range between 1 and 40 mg kg^−1^) (EPA, 2001) that possesses severe threats to all forms of life including plants, animals, and human beings (Hasanuzzaman et al., 2015; Chandrakar et al., 2018; Nahar et al., 2022). The level of As is affected by the increased anthropogenic activities, such as, extensive use of agrochemicals, fossil fuels, metal mining, smelting slags, and the disposal of industrial and municipal waste (Mehmood et al., 2017). Terrestrial arsenic is easily accessible and is taken up by plant roots, translocated into leaf tissue, and accumulated in the edible parts. This enables the entry of As into the food chain and causes serious threats to human health (Han et al., 2017). Moreover, high-arsenic concentration leads to several morpho-physiological, phenotypic, and genotypic damages in plants (Garg and Singla, 2011; Gusman et al., 2013; Li et al., 2023). The preliminary symptoms of As toxicity on plants are inhibition of seed germination, chlorosis, decrease in photosynthesis, transpiration rate, decrease root growth, yield, alteration in plant metabolism, and DNA damages that ultimately leads to reduced growth and productivity (Fatima et al., 2021b; Li et al., 2023).

Several approaches have been employed to neutralize/lessen the effect of As toxicity on plants. For instance, exogenous application of salicylic acid, silicon, and 24-Epi-Brassinolide has been shown to mitigate As stress in *Triticum aestivum* (Sil et al., 2018; Maghsoudi et al., 2020). Similarly, supplementation of melatonin-selenium nanoparticles, phosphorus, and silicon fertilizers detoxified As effects in *Brassica napus* (Farooq et al., 2022), and *Oryza sativa* (Li et al., 2009), respectively. In other studies plant growth promoting *Acinetobacter* sp. was observed to mitigate arsenic stress in *Cicer arietinum* (Srivastava and Singh, 2014).

Recent progress has been made to explore the potential of SMF to improve the development and stress tolerance of As-treated plants. The magnetopriming is a biophysical, non-invasive, environmental friendly method to promote plant growth and productivity in both normal and stressed environments (Sarraf et al., 2020). Studies reported that seeds primed with magnetic field prior to sowing promotes seed germination, root/shoot growth, seedling vigor, early growth characteristics, photosynthetic rate, and yield of various plants such as *Cicer arietinum* (Vashisth and Nagarajan, 2008), *Helianthus annuus* (Vashisth and Nagarajan, 2010), *Solanum lycopersicum* (Anand et al., 2019), *Zea mays* (Kataria et al., 2017a, 2019; Kataria et al., 2020a, b), and *Oryza sativa* (Florez et al., 2004). Involvement of magnetopriming in providing cellular defense against oxidative stress induced by HM toxicity, salinity, UV-B and drought has been reported in *Vigna radiata* (Chen et al., 2011), *Glycine max* (Baghel et al., 2016, Baghel et al., 2018; Kataria et al., 2017a, b; Kataria et al., 2020a, b; Prajapati et al., 2024), *Zea mays* (Kataria et al., 2017a) and *Cicer arietinum* (Thomas et al., 2013).

It is becoming increasingly evident that the uptake of HMs by roots seems to trigger a structural alteration in root system with potential functional consequences (Armendariz et al., 2016). Since roots are the primary tissues exposed to almost all kinds of soil stresses including metal toxicity and forced to modify their structural development accordingly. Previous research demonstrated the negative outcome of arsenic on root morphology and anatomy in *Raphanus sativus*, *Brassica oleracea*, *Brassica juncea* (de Freitas-Silva et al., 2016), and *Phaseolus aureus* (Singh et al., 2007). Arsenic toxicity has been found to decrease the root growth, disturbs root vascular cylinder diameter, and causes anatomical alterations like protoplast retraction, cell hypertrophy, cellular plasmolysis, and necrotic regions in plant roots (de Freitas-Silva et al., 2016). Plant roots absorb, uptake, and translocate water and minerals to the foliar tissues of the plants as well as serve as a bridge between plants and soil (Kul et al., 2020). Root system architecture (RSA; spatiotemporal configurations of roots) such as root hairs, main root growth, root length, branching, and lateral root development are the main components of root responsible for maintaining the nutritional status, growth, and development of plants under stress conditions (Karlova et al., 2021). Many studies have demonstrated the detrimental effects of arsenic on the growth of plant roots such as root length, root hair growth, and root toxicity index (Beniwal et al., 2023; Rakkammal et al., 2024). Investigating the RSA of plants under As toxicity can provide insight into the importance of root traits for abiotic stress tolerance. However, due to the limited capabilities of advanced techniques for observing roots, the effects of environmental stress on RSA have been less studied than the above ground parts of plants. In recent years’ synchrotron radiation (SR)–based techniques such as Fourier transform spectroscopy (SR-FTIR), x-ray fluorescence (SR-XRF), and x-ray micro-computed tomography (SR-µCT) have emerged as important tools to examine the structural and anatomical features of plant tissues including roots (Dhondt et al., 2010; Vijayan et al., 2015). In previous studies, x-ray micro-CT (a high-resolution three-dimensional imaging technique that provides qualitative and quantitative information on the structure of plant parts such as leaves, roots, and seeds) has been successfully used to investigate the alterations in leaf veins of soybean plants grown under UV-B stress, heavy metal, stress, and magnetoprimed conditions (Fatima et al., 2016, 2017, Fatima et al., 2021a, b). Therefore, it is possible to implicate x-ray micro-CT (SR-µCT) as an effective imaging technique to investigate the influence of magnetopriming on the RSA of arsenic stressed crop plants. Previously, magnetopriming induced alleviation of adverse effects of cadmium and mercury toxicity in soybean during seed germination and early seedling growth has been reported (Prajapati et al., 2024; Vyas et al., 2024). Magnetopriming has been shown to mitigate the detrimental effects of arsenic toxicity in soybean and Cd toxicity in mungbean on the photosynthetic rate and efficiency of PSII (Chen et al., 2011; Fatima et al., 2021b). However, the lessening of adverse effects of arsenic on RSA in soybean by magnetopriming has not been studied yet. It is therefore time for crop scientists to take advantage of the underutilized and underexplored range of RSA traits in order to assure stability and higher productivity in agricultural systems for future environmental conditions and climate change scenarios. *Glycine max* L. (soybean), the most important legume for nutrition, is used as a test crop in this study due to its high protein content and nutritional value. For this study, we hypothesized that soybean seeds treated with static magnetic field (SMF) before sowing, when exposed to arsenic toxicity, will change their root morphology to maintain the growth and productivity of the stressed plants. Therefore, the objectives of the present study were (i) to analyze the effect of magnetopriming on root length and root biomass of As-stressed plants and (ii) to observe the RSA of magnetoprimed As-stressed roots using x-ray micro-CT.

## Materials and methods

Soybean [*Glycine max* (L.) variety JS-9560] breeder seeds were procured from the Indian Institute of Soybean Research, Indore, India. The experiments were conducted in ambient conditions during September 2019 to November 2019 on the terrace of the School of Biochemistry, Devi Ahilya Vishwavidyalaya, Indore, India (latitude 2243′N). During the experiment’s period, the average temperature was between 27°C and 30°C, and the relative humidity ranged between 55% and 75%. Prior to sowing in the nursery bags, the SMF primed and non-primed (NP) seeds were mixed with 3g kg^−1^ *Rhizobium japonicum* strain (National Fertilizer Limited, New Delhi, India) and the recommended fungicides Bavistin and Dithiane Mat (2 g kg^−1^ seeds).

### Magnetic field generation and treatment

The fabricated electromagnetic field (EMF) generator (“AETec” Academy of Embedded Technology, Delhi, India) was designed with a 5-cm gap between its pole pieces. The generator comprises cylindrical pole pieces with a 9-cm diameter and a length of 16 cm. The coil within the generator is with 3,000 turns and possesses a resistance of 16 Ω. These electromagnets are connected to a direct current (DC) power supply with an output of 80 volts and 10 amperes, allowing for continuous adjustment of the current. To monitor the magnetic field strength within the pole gaps, a Gauss meter (DGM-30 by Testron Instruments) was utilize. The electromagnet coil current is regulated to achieve the desired magnetic field strength between the pole pieces. This EMF generator produce a magnetic field of strength ranging from 50 to 300 mT in a horizontal direction, as previously detailed in Kataria et al. (2020b) (**Supplementary Figure S1**). The sample holder of cylindrical shape made of nonmagnetic thin cardboard box (42 cm^3^ capacity) was kept between both the pole pieces. The SMF of 200 mT for 1h was applied for magnetopriming of the soybean seeds in present study. The dose was selected on the basis of our earlier research on magnetopriming of soybean seeds (Fatima et al., 2017).

The temperature around the seeds was sustained at 25°C ± 5°C throughout the treatment period. Similar seeds not exposed to magnetic field are served as NP seeds.

### Experimental setup and growth measurements

The seeds were germinated in nursery bags (34 cm H × 34 cm B) containing 5 kg of sand, black soil, and cow dung manure in the ratio of 1:2:1. Five to six seeds were germinated per bag and three bags are used for every treatment to arrange the experiment in completely randomized design. Arsenic in the form of sodium arsenate was added to the sand–soil mixture at four different levels (5, 10, and 50 mg As kg^−1^ soil) ranging from normal to arsenic polluted environment before seed germination. Sand–soil mixture without sodium arsenate treatment (0 mg As kg^−1^ of soil) was termed as control and used to compare the effects of arsenic toxicity in the plant emerged from NP seeds. The plants were irrigated regularly to avoid drought conditions. After 30 days of emergence of the seedlings; the plants were harvested for further study. The roots of the plants were separated from the aboveground parts. The length and weight of arsenic treated and control plants emerged from magnetoprimed, and NP seeds were measured in centimeters and grams using ruler and weighing balance.

### Tolerance index

% Tolerance index (in terms of root mass) of soybean plants emerged from NP and magnetoprimed seeds grown under different concentrations of arsenic toxicity were calculated with the formula given by Iqbal and Rahmati (1992).

### Synchrotron micro-computed tomography for root system architecture

The architecture of the root system, in particular the pore diameter, the growth of the main root, the development of the lateral roots, the branching of the roots, the formation of root nodules, and root hairs were investigated with SR-µCT. Advances in SR-µCT for research in various disciplines, including agriculture, have made it possible to characterize plant roots at the micrometer scale. SR-µCT enables 3D visualization and porosity characterization of plant roots in a non-destructive manner (Indore et al., 2022). The technique enables the visualization of roots without physical sections or staining compared to conventional methods (Keyes et al., 2013).

The experimental facility, Imaging Beamline (BL-4), synchrotron radiation source Indus-2, was used for the microcomputed tomographic examination of the samples (Fatima et al., 2021a, b). The synchrotron beam energy for root tomography is 10 keV and the CCD detector with a pixel size of 5 microns is used to obtain the 900 projections by rotating the sample in steps of 0.2° (Fatima et al., 2016). Cross-sectional images of the roots were created from the recorded projections using the Filtered Back Projection (FBP) reconstruction algorithm. Volume rendered images were created from the reconstructed slice stack for the roots using Drishti software (Limaye, 2012). The stack of reconstructed slices was denoised and post-processed further in the Fiji software. In order to quantify the pores visible in the root cross-sections, segmentation was performed to select the pores in the image stack and neglect the remaining part (Fatima et al., 2016).

### Statistical analysis

The experiments were conducted in a randomized block design with three biological replicates-3 nursery bags, were used for each treatment (5, 10, and 50 mg As kg^−1^ soil) and control (0 mg As kg^−1^ soil) for both SMF-primed (MP) and NP soybean seeds. The data representation form was mean ± SE (*n* = 3), taking five plants were in each replica for measurements of all the studied parameters. The data were evaluated by Student’s *t*-test, ^**^ *p* < 0.01; ^***^ *p* < 0.001 signify the difference amongst soybean plants originates from NP seeds grown under non-stressed and As toxicity conditions; ^##^ *p* < 0.01; ^###^ *p* < 0.001 signify the difference amongst NP and MP plants grown in non-stress and As toxicity conditions.

## Results and discussion

Arsenic is a non-essential noxious element for plants and animals. It is commonly present in the groundwater of heavily populated river deltas in Southeast Asian countries including India and used for irrigating agricultural crops. Arsenic from ground water is easily absorbed by plant roots and accumulates in these tissues, leading to constant changes in the architecture of the root system enable plants to survive in the polluted environment (Ronzan et al., 2018). Root architecture traits such as root diameter, length, density, branching, and nodulation are fundamental determinants for promoting robust plant growth under challenging environmental conditions. Improving these root traits has the potential to make a significant contribution to sustainable agricultural development and increased productivity, especially in the face of soil stress factors such as arsenic toxicity (Fenta et al., 2011).

**Figure 1:**
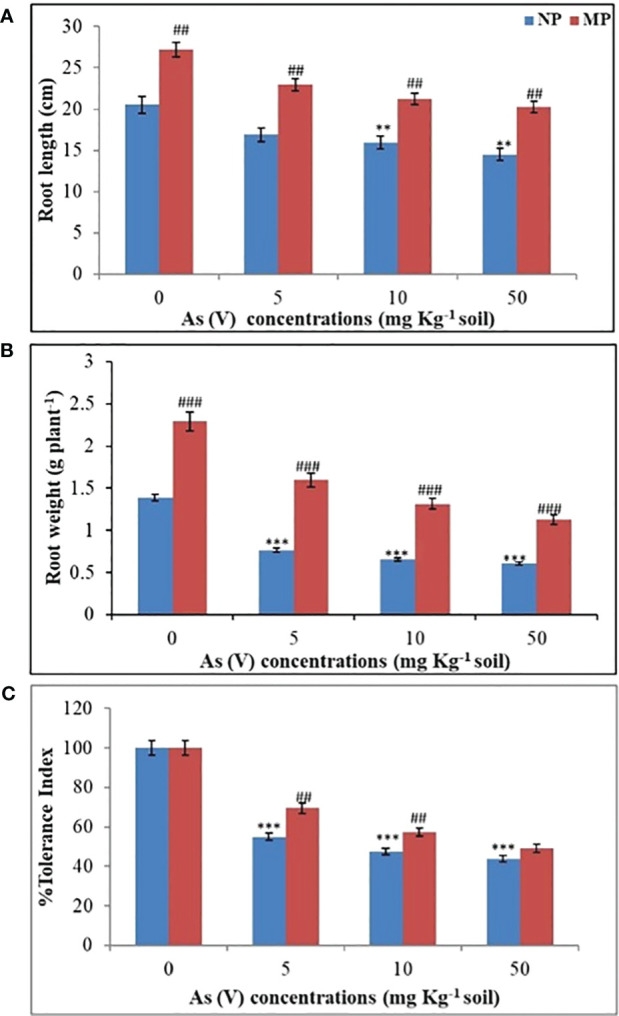
Effect of magnetopriming (200 mT for 1h) on root length **(A)**, root biomass **(B)** and % tolerance index **(C)** of soybean plants grown for 30 days in different level of As toxicity (0–50 mg kg^−1^ soil). The vertical lines on bar indicates ± S.E. for mean (*n* = 3). The data were evaluated by Student’s *t*-test, ^**^ *p* < 0.01; ^***^ *p* < 0.001 signify the difference among soybean plants originates from non-primed seeds grown under non-stressed and As-toxicity conditions; ^##^ *p* < 0.01; ^###^ *p* < 0.001 signify the difference among non-primed and MP plants grown in non-stress and As toxicity conditions. NP, non-primed and MP, magnetoprimed with SMF.

Our ongoing research is centered on magnetoprimed soybean plants and their adaptive responses to arsenic toxicity, with a specific emphasis on the development of root morphology. In present investigation, for the first time, we have employed advanced techniques like synchrotron micro-CT to quantify and analyze various root parameters, including root length, diameter, pore size, lateral root branching, and nodule formation in soybean plants. It is important to note that soybean exhibits a characteristic allorhizic root system, in which a tap root (originating from the hypocotyl) serves as the primary root, from which lateral roots subsequently emerge (Fenta et al., 2011). In the present study, we found significant reduction (*P* < 0.01) in the root length and root mass in NP plants exposed to different levels of As toxicity (5, 10, and 50 mg kg^−1^) in comparison to control (0 mg kg−^1^ soil) conditions (**Figures 1A, B**). The impact of arsenic on root growth, specifically in terms of both root length and root mass, became increasingly pronounced at higher arsenic concentrations. The maximum inhibition of 22% (10 mg kg^−1^ soil) and 29% (50 mg kg^−1^ soil) in root length (**Figure 1A**), and 53% (10 mg kg^−1^ soil) and 56% decrease (50 mg kg^−1^ soil) was observed in root biomass in soybean plants from NP seeds (**Figure 1B**) in comparison to control plants grown under non-stress conditions (0 mg kg^−1^ soil). A strong inhibition of root length at higher arsenic concentration were also previously reported by Rodríguez-Ruiz et al. (2019) in *Pisums ativum* and Singh et al. (2007) in *Phaseolus aureus*. Conversely, a progressive increase in the root length (33%) (**Figure 1A**) and root biomass (65%) (**Figure 1B**) was found in magnetoprimed plants in contrast to plants from NP seeds under control conditions. The use of SMF pretreatment resulted in a 36% increase in root length compared to plants from NP seeds when the soil contained 5 mg As per kg. Similarly, at higher arsenic concentrations of 10 and 50 mg As per kg in the soil, SMF pretreatment led to root length increases of 33% and 40%, respectively, compared to their corresponding plants from NP seeds (**Figure 1A**). Moreover, in response to As toxicity plants from magnetoprimed seeds showed 108%, 99%, and 86% improvement in root biomass as contrast to their corresponding NP ones, respectively, at 5, 10, and 50 mg As kg^−1^ soil (**Figure 1B**). The % tolerance index was determined based on root mass of seedlings that originated from both SMF-primed and NP seeds, in the presence or absence of As toxicity conditions (**Figure 1C**). The results revealed that as the concentration of As increased, the % tolerance index decreased in both NP and SMF-primed seedlings. Remarkably, seedlings from SMF-primed seeds consistently exhibited a higher % tolerance index compared to their respective NP counterparts across all tested concentrations of As, as depicted in **Figure 1C**. This illustrates that SMF pre-treatment to seeds abridged the phytotoxic effects of As on the roots through severely reducing the root mass. The magnetopriming positively influence on plant growth by stimulating root length and root biomass in plants under non-stress as well as abiotic stress factors such as salt and drought (Anand et al., 2012; Baghel et al., 2016, Baghel et al., 2018, 2019). Similarly, Galland and Pazur (2005) reported that MF pretreatment increased the resistance of plants to As toxicity by regulating the ionic flow in plant cell membranes. The comparable effects of magnetopriming were observed in soybean plants in promoting the growth of above ground parts, efficiency of PSII, photosynthesis, and water transport under arsenic stress (Fatima et al., 2021b). Root growth is a multifaceted process involving several important steps, including cell division in the root meristems, followed by differentiation and elongation of the descending cells (Beemster and Baskin, 1998). In particular, EMFs have been identified as an important factor responsible for promoting the development of metaxylem cells, which in turn contributes to an increased rate of root elongation (Bitonti et al., 2006). Consequently, the observed increase in root length resulting from SMF pre-treatment in our study may enhance the capacity for water and nutrient absorption, which is consistent with previous observations by Radhakrishnan and Kumari (2012).

Disturbances in plant–water relation are the primary effect of HM stress, which induces a series of changes in the whole plant. In roots, metal toxicity stimulates a reduction in water uptake and inhibits short distance water transport in the symplast and apoplast (Rucińska-Sobkowiak, 2016). Arsenic stress imbalances the water status in plants (Mubarak et al., 2016). For example, in response to arsenic stress, the substantial reduction in relative water content, stomatal conductance, water use efficiency and an increase in transpiration rate have been reported in *Triticum aestivum*, *Pisum sativum* (Garg and Singla, 2012, Hasanuzzaman and Fujita, 2013), *Glycine max* (Fatima et al., 2021b), and *Lactuca sativa* (Gusman et al., 2013).

**Figure 2:**
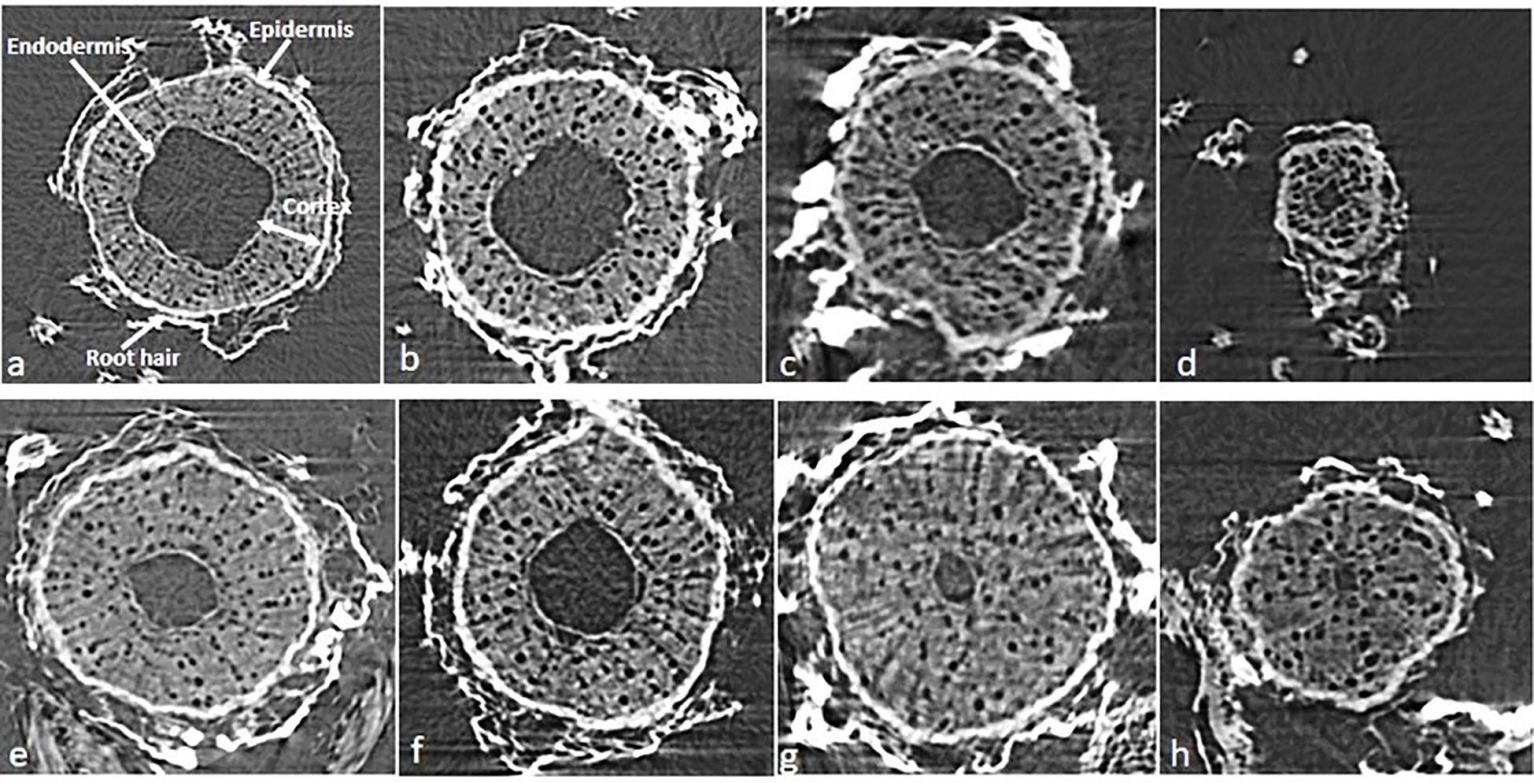
The cross-sectional images of tomography slices for roots of soybean plant (30-day old) grown from NP (upper row, **A**–**D**) and MP (lower row, **E**–**H**) seeds under different level of As toxicity (0–50 mg kg^−1^ soil), illustrates the pores in cortex region and the inner cylinder region of the roots. NP, non-primed and MP, magnetoprimed with SMF.

**Figure 3:**
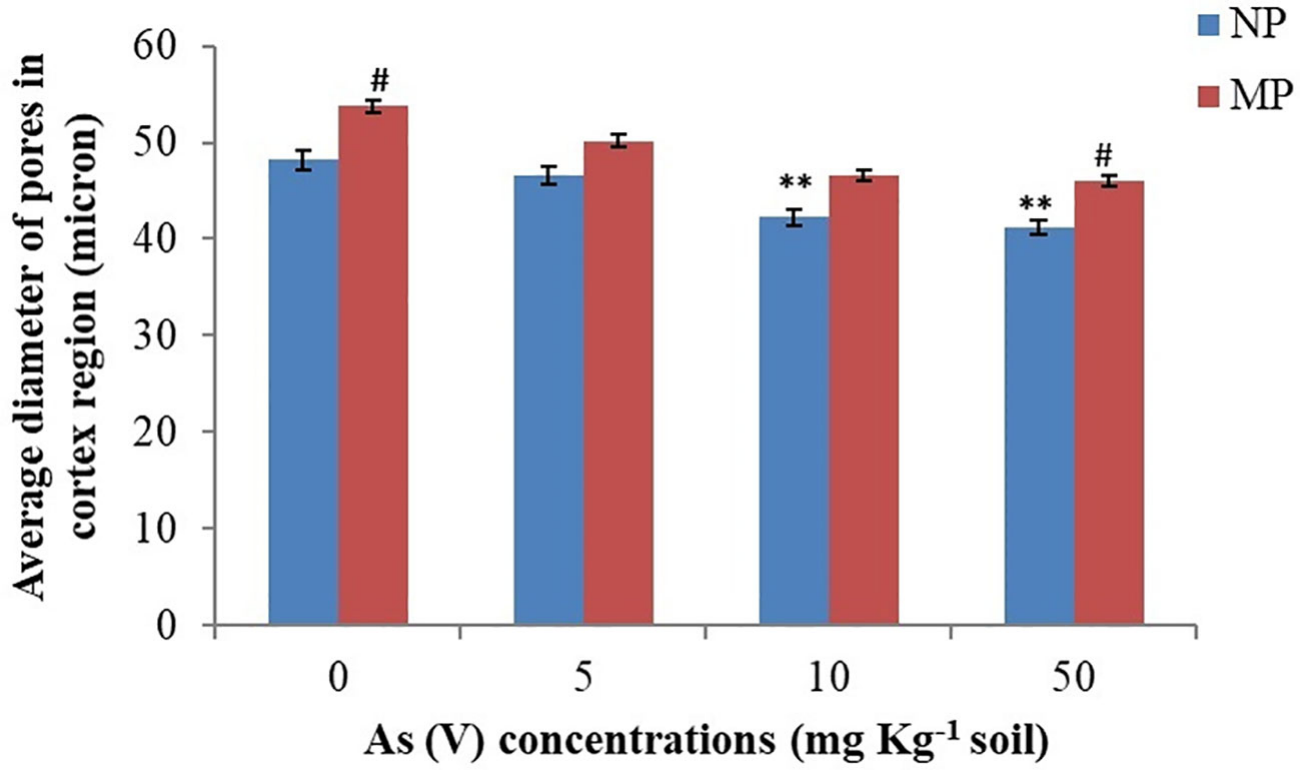
Effect of magnetopriming (200 mT for 1h) on diameter of pore size of cortical cells of roots in soybean plants grown for 30 days in different level of As toxicity (0–50 mg kg^−1^ soil). The vertical lines on bar indicates ± S.E. for mean (*n* = 3). The data was evaluated by Student’s *t*-test, ^**^ *p* < 0.01; signify the difference among soybean plants originates from non-primed seeds grown under non-stressed and As-toxicity conditions; ^#^ *p* < 0.05; signify the difference among non-primed and MP plants grown in non-stress and As toxicity conditions. NP, non-primed and MP, magnetoprimed with SMF.

**Figure 4:**
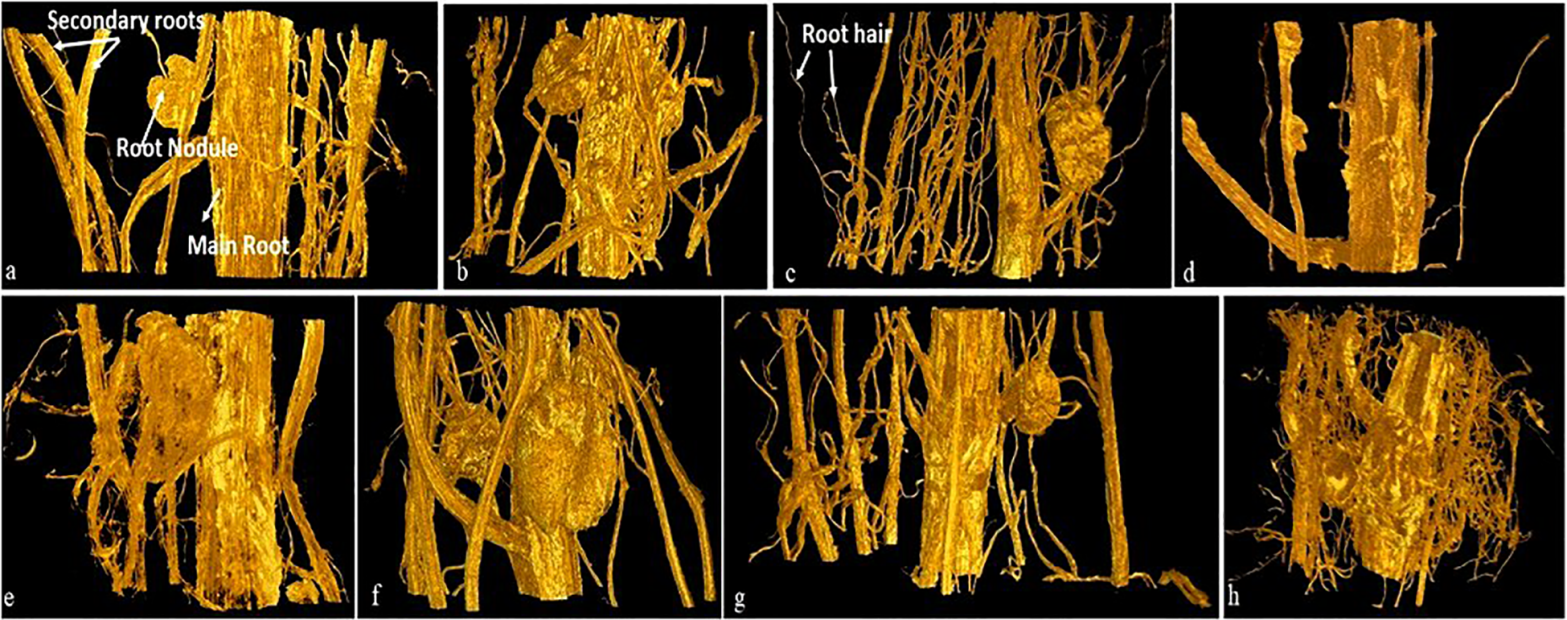
Volume rendered images of roots of soybean plant (30 day old) grown from NP (upper row, **A**–**D**) and MP (lower row, **E**–**H**) seeds under different level of As toxicity (0–50 mg kg^−1^ soil), represents main root, secondary root, root nodule and root hair. NP, non-primed and MP, magnetoprimed with SMF.

Impairment of water uptake by roots is related to the decrease in primary root length, root hair formation, and water absorbing area/root pore diameter by metal ions (Mahajan and Kaushal, 2018). Variations in root cortex pores diameter in response to arsenic stress are evident in the present study. Our results showed a substantial decrease in root hairs/complete absence of root hairs in the plants from NP seeds on arsenic exposure at higher concentration (50 mg As kg^−1^ soil) (**Figures 2B–D**) as compared to control plants where the root layers (epidermis, cortex and endodermis) were intact and root hairs were turgid (**Figure 2A**). A similar effect of As toxicity was observed when quantifying the diameter of the pores in the root cortex using the stack of tomography slices (**Figures 2**, **3**). Qualitative analysis of x-ray micro-CT images depicted that arsenic toxicity, damages the epidermal and cortex layer of root cells, losing their shape, size and showing the signs of shrinking and disintegration (**Figures 2C, D**). The pore diameter of cortical cells in NP plants decreases with increase in As concentration and the maximum pore size reduction was monitored at higher As treatment (50 mg kg^−1^ soil), which was 15% less as compared to control (0 mg kg^−1^ soil) (**Figure 3**). Similar alterations in root anatomy are observed in *Brasicca oleracea* and *Brasicca juncea* subjected to As toxicity (de Freitas-Silva et al., 2016). Pita-Barbosa et al. (2015) also found a reduction in the mitotic index of the apical meristem and parenchymal cell elongation in the As-treated seedlings, which led to uneven root cap growth and shorter roots as well as decrease in cellular gaps of cortex in *Cajanus cajan* roots. A microscopic analysis by Sofo et al. (2022) also revealed parallel distortion of the shape and conformation of root hairs in *Arabidopsis thaliana* after cadmium exposure. The root hairs of *A. thaliana* were strongly inhibited after Cd treatment (Sofo et al., 2022). However, the diameter of cortical cells pores in magnetoprimed plants was observed higher as compared to plants from NP seeds across all the tested concentrations of As (**Figures 2E–H**, **3**). Thus, the improvement in cortical pore size in magnetoprimed plants signifies the detoxification effects of magnetopriming against As stress. The SMF increases the root hydraulic activity of metal stressed plants and prevents them from water-stress induced by toxic metal ions. Our previous study on the effect of arsenic on leaf anatomy of magnetoprimed soybean plants supports the present results (Fatima et al., 2021b), as the hydraulic activity in plants comprises of the roots, stem and majorly the leaf midrib. Inhibition of primary root growth, alteration in lateral root density, decrease in number and size of nodules, are some common features of metal toxicity (van Dijk et al., 2022). HMs even at low concentrations damage and lower the density of lateral roots and root hairs (Baligar et al., 1998). In present study, the comparable results of arsenic toxicity on root traits are depicted in **Figure 4**. As shown in the figure, the root girth/thickness of primary root in NP plants (**Figures 4A–D**) decreases constantly with increase in arsenic concentration as compared to control plants. Moreover, arsenic decreased the volume of lateral roots, root hairs density, and nodules number in NP plants (**Figures 4A–D**). The highest reduction in lateral roots, root hairs, and nodule numbers were observed at the higher As concentration (50 mg kg^−1^ soil) (**Figure 4D**). Several research reported alike inhibitory effect of arsenic on nodule formation, root proliferation and extension as they are the primary tissues that come in direct contact with the metal ions (Kumar et al., 2020; Nahar et al., 2022). However, the magnetoprimed plants showed massive nodules and high number of root hairs at all the metal concentration (5, 10, and 50 mg As kg^−1^ soil) used including control as compared to NP plants (**Figures 4E–H**). Furthermore, the lateral roots in magnetoprimed plants are thicker in appearance than plants from NP seeds. This observation implies that magnetopriming neutralizes the phytotoxic effect of arsenic on root growth and development. Thus, the magnetopriming results in the improvement of RSA and tolerance of plants towards HM stress. The better root system may possibly enhanced nutrient and water uptake. Thus, SMF may increase the root hydraulic activity of arsenic stressed plants and prevent them from water-stress induced by toxic metal ions. It has been found previously that the magnetopriming improved the hydraulic activity that facilitates the transport of carbon, water, and nutrients in plants by increasing the average thickness of midrib and minor veins in soybean leaves (Fatima et al., 2021b). Some authors claim that magnetic fields may affect ion channel activation or ion transport within cells (García-Sancho and Javier, 1994; Galland and Pazur, 2005). Together, these factors contribute to the overall health and vigor of the plants ultimately leading to better yields under HM toxicity.

## Conclusion

The present study investigates the role of magnetopriming with SMF (200 mT for 1h) in alleviating the adverse effects of arsenic on RSA of soybean plants using synchrotron source base micro-computer tomography imaging technique. Arsenic vigorously inhibits the root growth and development by decreasing the root length, biomass, girth of primary root, root hairs formation, lateral roots branching, pore diameter of cortical cells, disintegrating the root layers and reducing the number and size of nodules. However, application of SMF treatment to soybean seeds prior to sowing results in the significant enhancement in root growth by improving the abovementioned root traits. Thus, our study concluded that magnetopriming has the potential to protect the plant roots from the adverse effect of As toxicity and enhance the tolerance of soybean plants against As toxicity. The present information on the impact of magnetopriming on existing plants under HM toxicity is quite less for its implication in the field conditions. Hence, future studies are needed on the detailed mechanism that how SMF priming is improving/altering the RSA, which signaling pathways/genes are activated by SMF? Further detailed studies need to be conducted to implement this technique with promising benefits in the field condition.

## Data availability statement

The original contributions presented in the study are included in the article/**Supplementary Material**. Further inquiries can be directed to the corresponding author.

## Author contributions

AF: Writing – review & editing, Conceptualization, Methodology, Writing – original draft, Data curation, Formal analysis, Funding acquisition, Investigation, Project administration, Resources, Software, Supervision, Validation, Visualization. SK: Conceptualization, Supervision, Writing – original draft, Writing – review & editing, Data curation, Formal analysis, Funding acquisition, Investigation, Methodology, Project administration, Resources, Software, Validation, Visualization. MJ: Writing – original draft, Supervision, Writing – review & editing, Data curation, Formal analysis, Funding acquisition, Investigation, Methodology, Project administration, Resources, Software, Validation, Visualization. RP: Writing – review & editing, Methodology, Writing – original draft, Data curation, Formal analysis. LM: Resources, Software, Validation, Visualization, Conceptualization, Funding acquisition, Supervision, Writing – original draft, Writing – review & editing, Formal analysis, Investigation, Project administration.

## Supplementary material

Supplementary Figure 1 Electromagnetic field generator for pre-treatment with static magnetic field (SMF) for 200 mT for 1h to soybean (*Glycine max*) variety JS-9560 seeds.
