# Supplementary figures and images for: Synchrotron tomography of magnetoprimed soybean plant root system architecture grown in arsenic-polluted soil
Source: Front Plant Sci. 2024 Jul 2;15:1391846. doi: 10.3389/fpls.2024.1391846 (PMC11249557; doi:10.3389/fpls.2024.1391846)

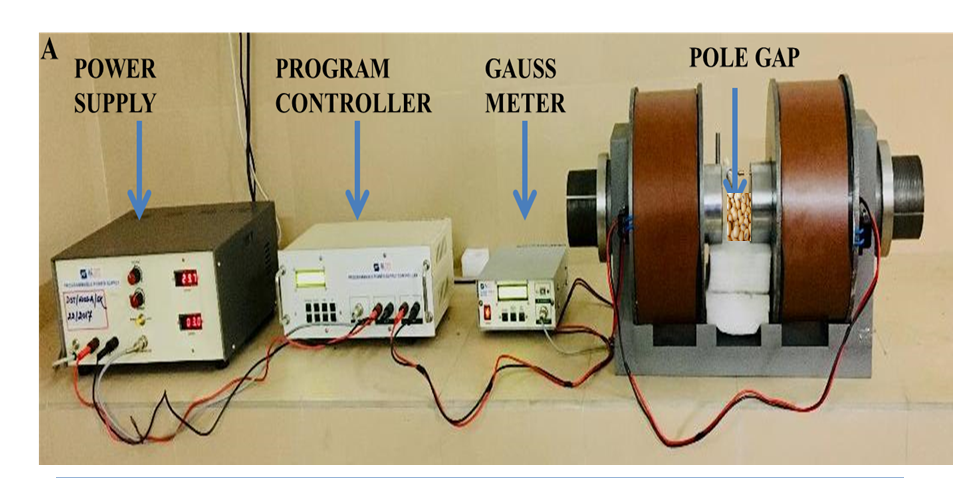

Supplement: Supplementary Figure 1 — Electromagnetic field generator for pre-treatment with static magnetic field (SMF) for 200 mT for 1h to soybean (Glycine max) variety JS-9560 seeds. [file Image_1.tif]
